# Neuronal localization of the 5-HT_2_ receptor family in the amygdaloid complex

**DOI:** 10.3389/fphar.2014.00068

**Published:** 2014-04-09

**Authors:** Cristiano Bombardi

**Affiliations:** Department of Veterinary Medical Sciences, University of BolognaBologna, Italy

**Keywords:** amygdaloid complex, pyramidal neurons, GABAergic interneurons, GABAergic projection neurons, serotonin, 5-HT_2A_ receptor, 5-HT_2B_ receptor, 5-HT_2*C*_ receptor

## Abstract

The amygdaloid complex (or amygdala), a heterogeneous structure located in the medial portion of the temporal lobe, is composed of deep, superficial, and “remaining” nuclei. This structure is involved in the generation of emotional behavior, in the formation of emotional memories and in the modulation of the consolidation of explicit memories for emotionally arousing events. The serotoninergic fibers originating in the dorsal and medial raphe nuclei are critically involved in amygdalar functions. Serotonin (5-hydroxytryptamine, 5-HT) regulates amygdalar activity through the activation of the 5-HT_2_ receptor family, which includes three receptor subtypes: 5-HT_2A_, 5-HT_2B_, and 5-HT_2*C*._ The distribution and the functional activity of the 5-HT_2_ receptor family has been studied more extensively than that of the 5-HT_2A_ receptor subtypes, especially in the deep nuclei. In these nuclei, the 5-HT_2A_ receptor is expressed on both pyramidal and non-pyramidal neurons, and could play a critical role in the formation of emotional memories. However, the exact role of the 5-HT_2A_ receptor subtypes, as well as that of the 5-HT_2B_ and 5-HT_2*C*_ receptor subtypes, in the modulation of the amygdalar microcircuits requires additional study. The present review reports data concerning the distribution and the functional roles of the 5-HT_2_ receptor family in the amygdala.

## Introduction

The amygdaloid complex (or amygdala), a heterogeneous structure located in the medial portion of the temporal lobe, is involved in multiple tasks, such as the generation of emotional behavior, formation of emotional memories related to fear and anxiety and modulation of the consolidation of explicit memories for emotionally arousing events (Aggleton, 2000; Whalen and Phelps, 2009). Several neuromodulators, including serotonin, are critical for amygdalar functions. Many neurological and psychiatric diseases, especially affective disorders, are characterized by a dysfunction of the amygdaloid complex and the serotoninergic system (Sanders and Shekhar, 1995; Jasnow and Huhman, 2001; Manji et al., 2001; Amaral, 2002; Braga et al., 2002; Hariri et al., 2002; Pralong et al., 2002; Rainnie et al., 2004; Canli et al., 2005; Keele, 2005; Kim et al., 2005; Rodrigues Manzanares et al., 2005; Hariri and Holmes, 2006; Shin et al., 2006; Van Nobelen and Kokkinidis, 2006). Selective serotonin reuptake inhibitors (SSRIs) are effective in the treatment of a variety of psychiatric diseases, such as anxiety disorders, in which the amygdaloid complex may play a role. Accordingly, it has been shown that the acquisition of auditory fear conditioning in the rat was enhanced by the SSRI citalopram when administered in acute cases and reduced when as administered in chronic cases; indeed, fear conditioning is known to be a model of emotional learning in which amygdaloid circuits play an important role (Burghardt et al., 2004). Selective serotonin reuptake inhibitors also reduce conditioned fear through its effect on the amygdala (Inoue et al., 2004). Moreover, co-administration of serotonin receptor agonists with paroxetine and venlafaxine could enhance the therapeutic effects of these drugs (Dhonnchadha et al., 2005).

## Anatomical organization and major cell types of the amygdala

**Figure 1:**
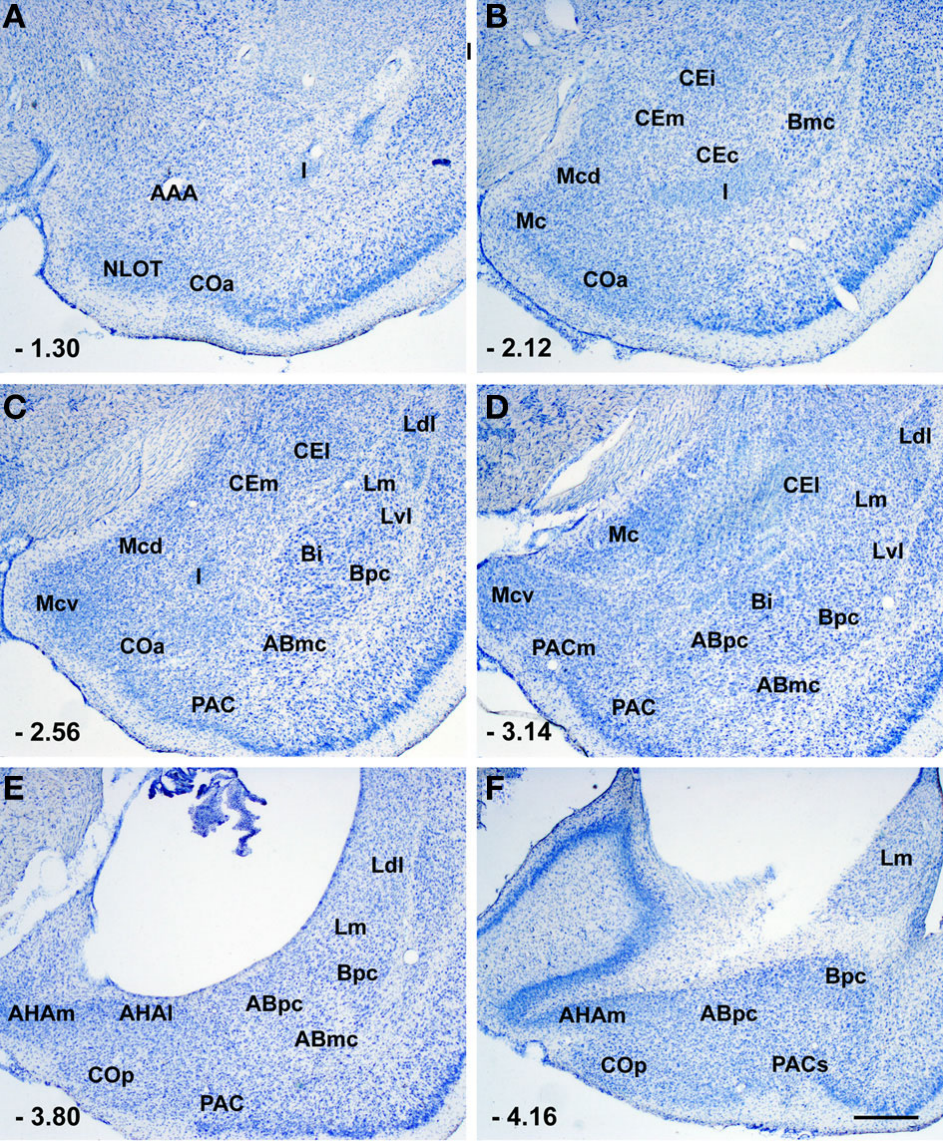
**Photomicrographs from thionin-stained coronal sections of the rat amygdala showing the various nuclei and nuclear subdivision**. Scale bar = 500 μm in **(F) (**applied to **A–F)**. For abbreviations see Table 1. The numbers in the lower left corner correspond to the distance from bregma according to rat brain atlas of Paxinos and Watson (1998).

The amygdala is composed of numerous nuclei and areas with different cytoarchitectonic, chemoarchitectonic, and connectional characteristics. In particular, this structure is composed of deep, superficial, and “remaining” nuclei (or areas) (Pitkänen, 2000; Pitkänen and Kemppainen, 2002). The deep nuclei include the lateral nucleus, the basal nucleus, the accessory basal nucleus, and the paralaminar nucleus (especially in primates). The lateral, basal, and accessory basal nuclei constitute the basolateral amygdala. The superficial nuclei include the anterior cortical nucleus, the nucleus of the lateral olfactory tract, the bed nucleus of the accessory olfactory tract, the medial nucleus and the posterior cortical nucleus. The remaining nuclei consist of the anterior amygdaloid area, the central nucleus, the intercalated nuclei and the amygdalohippocampal area (Pitkänen, 2000; Pitkänen and Kemppainen, 2002). Each nucleus can be partitioned into different subdivisions, as reported in Table 1 and Figure 1.

**Table 1:** **Nuclei and nuclear subdivisions of the rat, the monkey and the human amygdala (modified from Pitkänen and Kemppainen, 2002)**.

**Nuclei**	**Rat subdivisions**	**Monkey subdivisions**	**Human subdivisions**
Lateral nucleus (L)	Dorsolateral (Ldl)	Dorsal	Lateral
	Medial (Lm)	Dorsal intermediate	Medial
	Ventrolateral (Lvl)	Ventral intermediate	
		Ventral	
Basal nucleus (B)	Magnocellular (Bmc)	Magnocellular	Magnocellular
	Intermediate (Bi)	Intermediate	Intermediate
	Parvicellular (Bpc)	Parvicellular	Parvicellular
Accessory basal nucleus (AB)	Magnocellular (ABmc)	Magnocellular	Magnocellular
	Parvicellular (ABpc)	Parvicellular	Parvicellular
		Ventromedial	Ventromedial
Paralaminar nucleus	Absent	No subdivisions	Lateral
			Medial
Bed nucleus of the accessory olfactory tract	No subdivisions	Absent	Absent
Medial nucleus (M)	Rostral (Mr)	No subdivisions	No subdivisions
	Central dorsal (Mcd)		
	Central ventral (Mcv)		
	Caudal (Mc)		
Nucleus of the lateral olfactory tract (NLOT)	No subdivisions	No subdivisions	No subdivisions
Anterior cortical nucleus (COa)	No subdivisions	No subdivisions	No subdivisions
Periamygdaloid cortex (PAC)	Periamygdaloid cortex (PAC)	PAC oral	PAC oral
	PAC medial (PACm)	PAC1	PAC1
	PAC sulcal (PACs)	PAC2	PAC3
		PAC3	PAC sulcal
		PAC sulcal	
Posterior cortical nucleus (COp)	No subdivisions	No subdivisions	No subdivisions
Anterior amygdaloid area (AAA)	No subdivisions	No subdivisions	No subdivisions
Central nucleus (CE)	Capsular (CEc)	Lateral	Lateral
	Lateral (CEl)	Medial	Medial
	Intermediate (CEi)		
	Medial (CEm)		
Intercalated nuclei (I)	No subdivisions	No subdivisions	No subdivisions
Amygdalohippocampal area (AHA)	Lateral (AHAl)	Dorsal	Lateral
	Medial (AHAm)	Ventral	Medial

The deep nuclei consist of two types of neurons: excitatory (glutamatergic) pyramidal cells and inhibitory (GABA[γ-aminobutiric acid]ergic) non-pyramidal neurons (McDonald, 1992, 1998; Sah et al., 2003; Spampanato et al., 2011). Pyramidal cells have spiny dendrites, form nearly 80% of the total cell population, and act as projection neurons (McDonald, 1992, 1998; Sah et al., 2003). Non-pyramidal neurons are spine-sparse or aspiny cells, represent approximately 20% of all neurons, and constitute local circuits. These cells have axon collaterals restricted to the deep nuclei, acting as interneurons (McDonald, 1992, 1998; Sah et al., 2003; Spampanato et al., 2011). However, recent tracing studies combined with immunohistochemistry have demonstrated that some GABAergic neurons in the deep nuclei originate long projections directed to the prefrontal cortex (McDonald, 1987), basal forebrain (McDonald et al., 2012) and mediodorsal thalamic nucleus (McDonald, 1987; McDonald and Mascagni, 2007). As in the neocortex and hippocampal region, interneurons in the deep nuclei can be classified into distinct subpopulations on the basis of their content of calcium binding proteins (calbindin-D28k [CB], parvalbumin [PV] and calretinin [CR]), and peptides (somatostatin [SOM], cholecystokinin [CCK], neuropeptide Y [NPY], and vasoactive intestinal peptides [VIP]). The CB- and CR-immunoreactive (IR) interneurons are the predominant interneuronal subpopulations in the deep nuclei (Kemppainen and Pitkänen, 2000; McDonald and Mascagni, 2001). The CB-IR neurons can express PV (CB+/PV+) or CCK (CB+/CCK+) or SOM (CB+/SOM+) (McDonald and Betette, 2001; McDonald and Mascagni, 2002; Mascagni and McDonald, 2003). Similarly, the CR-IR interneurons can be immunopositive for CCK and/or VIP (Mascagni and McDonald, 2003; Muller et al., 2003). Microcircuits located in the deep nuclei are tightly regulated through the activity of the interneurons, which can innervate pyramidal cells as well as other interneurons. In particular PV-IR interneurons form symmetrical synapses with perisomatic (cell body, axon initial segment and thick proximal dendrites) and distal dendritic (small-caliber dendrites and dendritic spines) domains of pyramidal cells (Muller et al., 2006). Interestingly, pyramidal cells constitute synapse-like contacts with the perisomatic and distal dendritic domains of the PV-IR interneurons, in this way constituting a reciprocal connection (McDonald et al., 2005). The PV-IR interneurons also form interneuronal networks interconnected by electrical (gap junctions) and chemical synapses (Muller et al., 2005; Woodruff and Sah, 2007). PV-interneurons of the basolateral amygdala can be subdivided into four functionally distinct subpopulations. Fast spiking cells are the most common PV-IR functional cell type. Interestingly, these cells innervate the perisomatic domain of pyramidal neurons (Woodruff and Sah, 2007). The CCK-IR interneurons form synapses with the somata and the proximal dendrites of the pyramidal cells. The SOM-IR neurons provide an inhibitory innervation (symmetrical synapses), especially of the distal dendritic domain (small-caliber dendrites and dendritic spines) of pyramidal cells (Muller et al., 2007a). In addition, SOM-IR axon terminals also contact SOM-, VIP- and PV-IR interneurons (Muller et al., 2007a). The VIP-IR interneurons do not innervate pyramidal cells, but form synapses with other interneurons, especially CCK-positive interneurons (Mascagni and McDonald, 2003; Muller et al., 2003).

The superficial nuclei exhibit two major cell classes: spiny pyramidal cells and spine-sparse or aspiny non-pyramidal neurons (McDonald, 1992, 1998; Sah et al., 2003). Pyramidal cells are glutamatergic projection neurons whereas non-pyramidal neurons represent local inhibitory GABAergic interneurons. These cells are not randomly organized, as in the deep nuclei, but exhibit a laminar organization (layers I, II and III) (McDonald, 1992, 1998; Sah et al., 2003). Interestingly, GABAergic projection neurons are also distributed in the superficial nuclei (McDonald et al., 2012). The medial nucleus does not contain pyramidal and non-pyramidal neurons but small- to medium-sized ovoid neurons which possess spiny dendrites (McDonald, 1992, 1998; Sah et al., 2003).

In the amygdalohippocampal area, pyramidal and non-pyramidal neurons similar to those located in the deep nuclei are the two main cell types (McDonald, 1992, 1998). Similarly, the anterior amygdaloid area contains spiny projection neurons and aspiny interneurons (McDonald, 1992, 1998). Central nucleus and intercalated masses exhibits striatal-like GABAergic neurons (McDonald, 1992, 1998; Sah et al., 2003). Moreover, neurons located in the central nucleus can be subdivided into distinct subpopulations based on their expression of neuropeptides (neurotensin, corticotropin-releasing factor, enkephalin, galanin, SOM, substance P, CCK, and VIP) (Cassell et al., 1986; Cassell and Gray, 1989).

## Serotoninergic innervation of the amygdala and serotonin receptor

Serotonin (5-hydroxytryptamine, 5-HT) is a molecule located in the central nervous system which has the role of a neurotransmitter/neuromodulator. Serotoninergic somata are located along the midline of the brainstem in cell body groups designated raphe nuclei. The amygdala receives substantial serotoninergic innervation originating mainly from the dorsal raphe nucleus and, to a lesser extent, from the median raphe nucleus (Pralong et al., 2002; Hensler, 2006; Asan et al., 2013). Within the rat amygdala, serotoninergic fibers are directed especially in the lateral nucleus, basal nucleus (magnocellular division) and amygdalohippocampal area (Steinbusch, 1981). On the contrary, in the monkey amygdala, the highest density of serotoninergic fibers is located in the central nucleus, the nucleus of the lateral olfactory tract, the paralaminar nucleus, the anterior amygdaloid area, and the amygdalohippocampal area (Bauman and Amaral, 2005). In the rat basal nucleus (magnocellular and intermediate divisions), an ultrastructure study has demonstrated that serotonin terminals contact pyramidal as well as non-pyramidal (PV-IR and VIP-IR) neurons (Muller et al., 2007b).

The different physiological effects of serotonin are mediated by seven families of receptors (5-HT_1_–5-HT_7_). With the exception of the 5-HT_3A_/_3B_ receptors, which are a ligand-gated ion channel, the serotonin receptors are metabotropic receptors and belong to the G-protein coupled receptor (GPCR) superfamily (Barnes and Sharp, 1999; Hoyer et al., 2002). The 5-HT2 receptor family contains three receptor subtypes, 5-HT2A (471 amino acids), 5-HT2B (479–504 amino acids) and 5-HT2C (458–460 amino acids), which exhibit a 46–50% overall sequence identity and couple preferentially to Gq/11 to increase the hydrolysis of inositol phosphates and elevate intracellular calcium. 5-HT2 receptors may also couple to G12/13 which are known to mediate long term structural changes in cells (Barnes and Sharp, 1999; Hoyer et al., 2002; Hannon and Hoyer, 2008).

The 5-HT2A receptor is coupled to G-protein and stimulates phosphoinositide-specific phospholipase C with a consequent increment of inositol triphosphate (Raymond et al., 2001; Hoyer et al., 2002; Hannon and Hoyer, 2008). This serotonin receptor also activates phospholipase D and phospholipase A2 by interacting with additional G-proteins. The 5-HT2A receptor activation also closes potassium channels, producing neuronal depolarization (Aghajanian, 1995; Barnes and Sharp, 1999). In addition, the activations of this receptor subtype increases also cGMP levels by means of a mechanism dependent on N-methyl-D-aspartate (NMDA) receptor activation (Regina et al., 2003, 2004). Interestingly, the 5-HT2A and 5-HT2C receptors are paradoxically regulated by agonists and antagonists (Gray and Roth, 2001; Van Oekelen et al., 2003).

## Expression of 5-HT_2_ receptor subtypes in the amygdaloid complex

The amygdaloid complex expresses moderate to high density of serotonergic receptors including 5-HT1A, 5-HT2, 5-HT3, 5-HT4, and 5-HT6 (Pralong et al., 2002).

Using autoradiography, *in situ* hybridization and immunohistochemistry, it has been demonstrated that 5-HT_2_ receptor family mRNA and protein are present in the amygdala. Interestingly, the expression of the 5-HT_2A_ and 5-HT_2C_ receptors varied during postnatal development in the rat amygdaloid complex (Li et al., 2004).

### Deep nuclei

An autoradiographic study has demonstrated a 5-HT_2_ receptor binding site in rat deep nuclei, especially in the lateral nucleus (Pazos et al., 1985). In rodents, the presence of the 5-HT_2_ receptor in the lateral, basal and accessory basal nuclei was also verified with *in situ* hybridization experiments (Wright et al., 1995). Autoradiography and *in situ* hybridization studies have reported that binding sites and 5-HT_2A_ receptor mRNA are present in the lateral (dorsomedial division) and basal (magnocellular division) nuclei (Lopez-Gimenez et al., 2001). Pompeiano et al. (1994) have reported the presence of the 5-HT_2C_ receptor mRNA in rat deep nuclei, with the highest levels in the lateral nucleus. Interestingly, these Authors failed to find 5-HT_2A_ receptor mRNA in the same nuclei. Radioactive *in situ* hybridization studies on the rat (Greenwood et al., 2012) and the mouse (Li et al., 2003) amygdala have shown that the 5-HT_2C_ receptor mRNA is located in the lateral nucleus and, to a lesser extent, in the basal nucleus. Using non-radioactive *in situ* hybridization procedures, the highest number of cells containing 5-HT_2C_ receptor mRNA in the rat amygdala has been observed in the lateral and accessory basal nuclei. On the contrary, only a few 5-HT_2C_ receptor mRNA-reactive cells have been reported in the rat basal nucleus (Bonn et al., 2012, 2013). 5-HT_2C_ receptor mRNA has been reported in the deep nuclei of the human amygdala (Pasqualetti et al., 1999).

In rat deep nuclei, immunoreactivity for the 5-HT_2A_ receptor is located in pyramidal and non-pyramidal neurons (Morilak et al., 1993; Cornea-Hébert et al., 1999; Xu and Pandey, 2000; McDonald and Mascagni, 2007; Jiang et al., 2008; Bombardi, 2011; Bombardi and Di Giovanni, 2013). In the rat, 100% of the pyramidal cells express the 5-HT_2A_ receptor (McDonald and Mascagni, 2007; Bombardi, 2011). The 5-HT_2A_ receptor is abundant in the apical dendrites of pyramidal cells (McDonald and Mascagni, 2007; Bombardi, 2011) where it may amplify the impact of excitatory synaptic currents.

In rat deep nuclei, 5-HT_2A_ receptor immunoreactivity has been observed in GABAergic interneurons (in somata and dendrites) and GABAergic projection neurons (Morilak et al., 1993; McDonald and Mascagni, 2007; Bombardi, 2011). The GABAergic interneurons are present in the lateral, basal and accessory basal nuclei where the 5-HT_2A_ receptor is expressed by 66.3, 70.6, and 66.4% of interneurons, respectively (Bombardi, 2011). These interneurons are particularly abundant in the medial subdivision of the lateral nucleus (74.7% of interneurons) and in the parvicellular and magnocellular subdivisions of the basal nucleus (73.8 and 71.9% of interneurons, respectively) (Bombardi, 2011). In the rat amygdala, 59.8% of PV-IR neurons in the medial subdivision of the lateral nucleus, and 75.6% of PV-IR neurons in the magnocellular subdivision of the basal nucleus exhibit the 5-HT_2A_ receptor (McDonald and Mascagni, 2007). On the contrary, only 33.1% of SOM-IR neurons in the lateral nucleus (medial subdivision), and 32.6% of SOM-IR neurons in the basal nucleus (magnocellular subdivision), express the 5-HT_2A_ receptor (McDonald and Mascagni, 2007). The GABAergic/5-HT_2A_ receptor-IR projection neurons are especially distributed near the external and internuclear borders of the rat basolateral amygdala and project to the mediodorsal thalamus (McDonald and Mascagni, 2007).

5-HT_2C_ receptor-IR neurons, possibly pyramidal cells, have been observed in rat lateral and basal nuclei (Clemett et al., 2000).

#### Superficial nuclei

In the rat, 5-HT_2_ receptor mRNA levels are moderate in every superficial nuclei (Wright et al., 1995). In rat superficial nuclei, 5-HT_2A_ receptor mRNA is detectable only in the bed nucleus of the accessory olfactory tract where it is strongly expressed (Pompeiano et al., 1994). On the contrary, 5-HT_2C_ receptor mRNA is located in different superficial nuclei, such as the anterior cortical nucleus, the bed nucleus of the accessory olfactory tract and the medial nucleus. In particular, 5-HT_2C_ receptor mRNA levels are high in the bed nucleus of the accessory olfactory tract, intermediate in the medial nucleus and low in the anterior cortical nucleus (Pompeiano et al., 1994). Autoradiographic analyses of the rat brain have demonstrated the presence of the 5-HT_2_ receptor binding sites especially in the anterior cortical nucleus, but also in other superficial nuclei (Pazos et al., 1985). Immunoreactivity for the 5-HT_2A_ receptor has been observed in every superficial nucleus of the rat amygdala. However, a high density of immunopositive neurons is present, especially in the nucleus of the lateral olfactory tract and in the bed nucleus of the accessory olfactory tract (Morilak et al., 1993; Cornea-Hébert et al., 1999; Bombardi, 2011). Using *in situ* hybridization procedures, high levels of 5-HT_2C_ receptor mRNA have been observed in the medial nucleus and in the anterior cortical nucleus of the rodent amygdala (Li et al., 2003; Bonn et al., 2012, 2013; Greenwood et al., 2012). Accordingly, many 5-HT_2*C*_ receptor-IR neurons are located in the rat medial nucleus (Clemett et al., 2000). A moderate level of 5-HT_2C_ receptor-IR neurons has also been observed in the posterior cortical nucleus of the rat amygdala (Clemett et al., 2000).

As in the deep nuclei, the 5-HT_2A_ receptor is also expressed in pyramidal and non-pyramidal neurons in the rat superficial nuclei (Bombardi, 2011). Pyramidal cells are especially distributed in the nucleus of the lateral olfactory tract (layer II), the anterior cortical nucleus (layers II and III), the periamygdaloid cortex (layers II and III) and the posterior cortical nucleus (layers II and III) (Bombardi, 2011). In these cells, the 5-HT_2A_ receptor is strongly expressed in the apical dendrites where it may induce excitatory synaptic currents. The 5-HT_2A_ receptor-IR non-pyramidal neurons are distributed in the nucleus of the lateral olfactory tract, the anterior cortical nucleus, the periamygdaloid cortex and the posterior cortical nucleus. These cells are heterogeneous in shape (multipolar and fusiform) and size (from small to large), and are particularly abundant in layers II and III (Bombardi, 2011). Since the cell types in the medial nucleus are not cortex-like as in the other superficial nuclei, 5-HT_2A_ receptor-IR pyramidal and non-pyramidal neurons are not present in this nucleus (McDonald, 1992, 1998; Sah et al., 2003). Accordingly, the rat medial nucleus contains 5-HT2AR-IR principal neurons with ovoid cell bodies (Bombardi, 2011). The rat medial nucleus is the only amygdaloid area containing 5-HT_2B_ receptor-IR neurons. These cells are numerous and show a multipolar and bipolar morphology (Duxon et al., 1997a).

#### Remaining nuclei

In the rat amygdalohippocampal area, the presence of the 5-HT_2A_ receptor has been demonstrated only with immunohistochemical procedures which have revealed many 5-HT_2A_ receptor-IR neurons with angular- and ovoid-shaped cell bodies (Bombardi, 2011). The rat amygdalohippocampal area also contains a high density of 5-HT_2C_ receptor-IR neurons (Clemett et al., 2000). A moderate level of 5-HT_2A_ receptor mRNA has been revealed in the rat central nucleus (Wright et al., 1995). Accordingly, immunohistochemical procedures have demonstrated the presence of many 5-HT_2A_ receptor-IR ovoid somata in the different subdivisions of the rat central neurons (Cornea-Hébert et al., 1999; Bombardi, 2011). These cells could be GABAergic local neurons as well as GABAergic projecting neurons (Bombardi, 2011). Cells containing 5-HT_2C_ receptor mRNA have been observed in the rat central nucleus where they are particularly numerous in the lateral capsular subdivision (Bonn et al., 2012, 2013). Both 5-HT_2A_ and 5-HT_2C_ receptor mRNA are present at low density in the rat amygdalohippocampal area. Accordingly, pyramidal and non-pyramidal neurons of the rat amygdalohippocampal area contain the 5-HT_2A_ receptor (Bombardi, 2011). In the rat intercalated nuclei, 5-HT_2C_ receptor mRNA is present at a low density while 5-HT_2A_ receptor mRNA has not been detected (Pompeiano et al., 1994). A different distribution of the 5-HT_2A_ receptor has been observed using immunohistochemical procedures. In fact, Xu and Pandey (2000), and Bombardi (2011) have observed that small and large neurons in the rat intercalated nuclei express the 5-HT_2A_ receptor. The rat intercalated nuclei contain only weak 5-HT_2*C*_ receptor mRNA-reactive cells (Bonn et al., 2012, 2013). These data are in disagreement with immunohistochemical studies showing that intercalated nuclei contain a high density of 5-HT_2C_ receptor-IR neurons (Clemett et al., 2000).

## Effect of 5-HT_2_ receptor family activation on amygdalar neurons and microcircuits

Serotonin influences amygdalar information processing by activating multiple 5-HT_2_ receptor subtypes. Inasmuch as the amygdaloid microcircuits are complex and the expression patterns of the 5-HT_2_ receptor subtypes are not fully characterized, the mechanisms by which 5-HT_2_ receptor subtypes modulate amygdalar neurotransmission remains poorly understood. This modulation is complex and has been studied especially for 5-HT_2A_ and 5-HT_2C_ receptors.

Electrophysiological studies have demonstrated that the 5-HT_2A_ receptor activates the pyramidal cells of the deep nuclei. In fact, the local injection of 1-(2,5-dimethoxy-4-iodophenyl)-2-aminopropane (DOI), a 5-HT_2A_/5-HT_2C_ receptors agonist, increases the discharge rate (Stein et al., 2000) and facilitates synaptic plasticity via an NMDA-mediated mechanism (Chen et al., 2003) in presumptive pyramidal neurons of the rat basolateral amygdala.

The 5-HT_2_ receptor family also modulates the excitability of GABAergic interneurons in the deep nuclei. In fact, electrophysiological studies have demonstrated that the application of α-methyl-5-hydroxytryptamine (a 5-HT_2_ receptor agonist) and DOI (a 5-HT_2A_/5-HT_2C_ receptor agonist), induces the activation of GABAergic interneurons of the rat basolateral amygdala (Rainnie, 1999; Stein et al., 2000; Sokal et al., 2005). In addition, the stimulation of the 5-HT_2A_ receptor increases the frequency and amplitude of spontaneous inhibitory postsynaptic currents (sIPSCs) recorded from the pyramidal neurons of the juvenile rat basolateral amygdala (Jiang et al., 2008). Accordingly, the inhibition of pyramidal cell firing in the lateral nucleus of the rat amygdala obtained after the local application of serotonin is blocked by the simultaneous application of bicuculline methiodide, a GABA antagonist (Stutzmann and LeDoux, 1999). The activation of GABAergic interneurons of the corticomedial amygdala has been demonstrated by iontophoretic injections of DOI (Stein et al., 2000).

**Figure 2:**
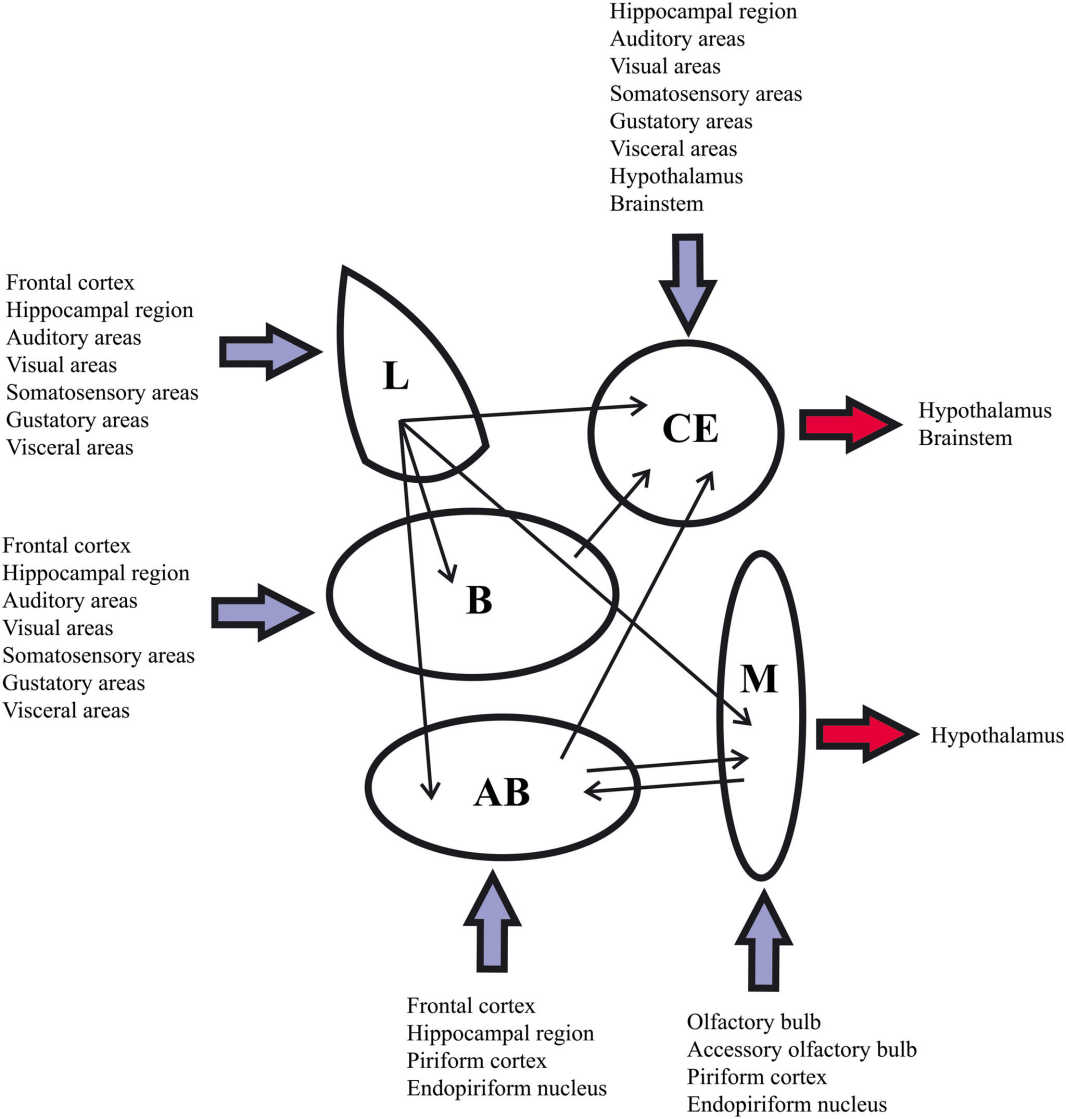
**Summary of the main extra-amygdaloid and intra-amygdaloid connections in the rat**. For abbreviations see Table 1.

Amygdala microcircuitry has not been studied as extensively as that of the neocortex and hippocampal region. However, numerous studies report that the amygdala circuit organization combines cortex-like (deep nuclei, the majority of the superficial nuclei and the amygdalohippocampal area) and striatum-like structures (central nucleus and intercalated nuclei) (McDonald, 1992, 1998; Sah et al., 2003). Since these amygdaloid areas provide numerous intra-amygdaloid and extra-amygdaloid connections, the amygdala is considered to be the interface of the information exchange between the various functional systems of the brain (Pitkänen, 2000). Traditionally, the extra-amygdaloid afferents (all the modalities of sensory inputs and polymodal inputs) target the input side of the amygdala (deep and superficial nuclei) where they are processed locally and then directed, by intra-amygdaloid connections, to the medial and central nuclei which act as an output station. The medial nucleus especially projects to the hypothalamic neuroendocrine zone whereas outputs from the central nucleus especially innervate the hypotalamic and brainstem nuclei which regulate autonomic functions (Pitkänen, 2000; Figure 2).

**Figure 3:**
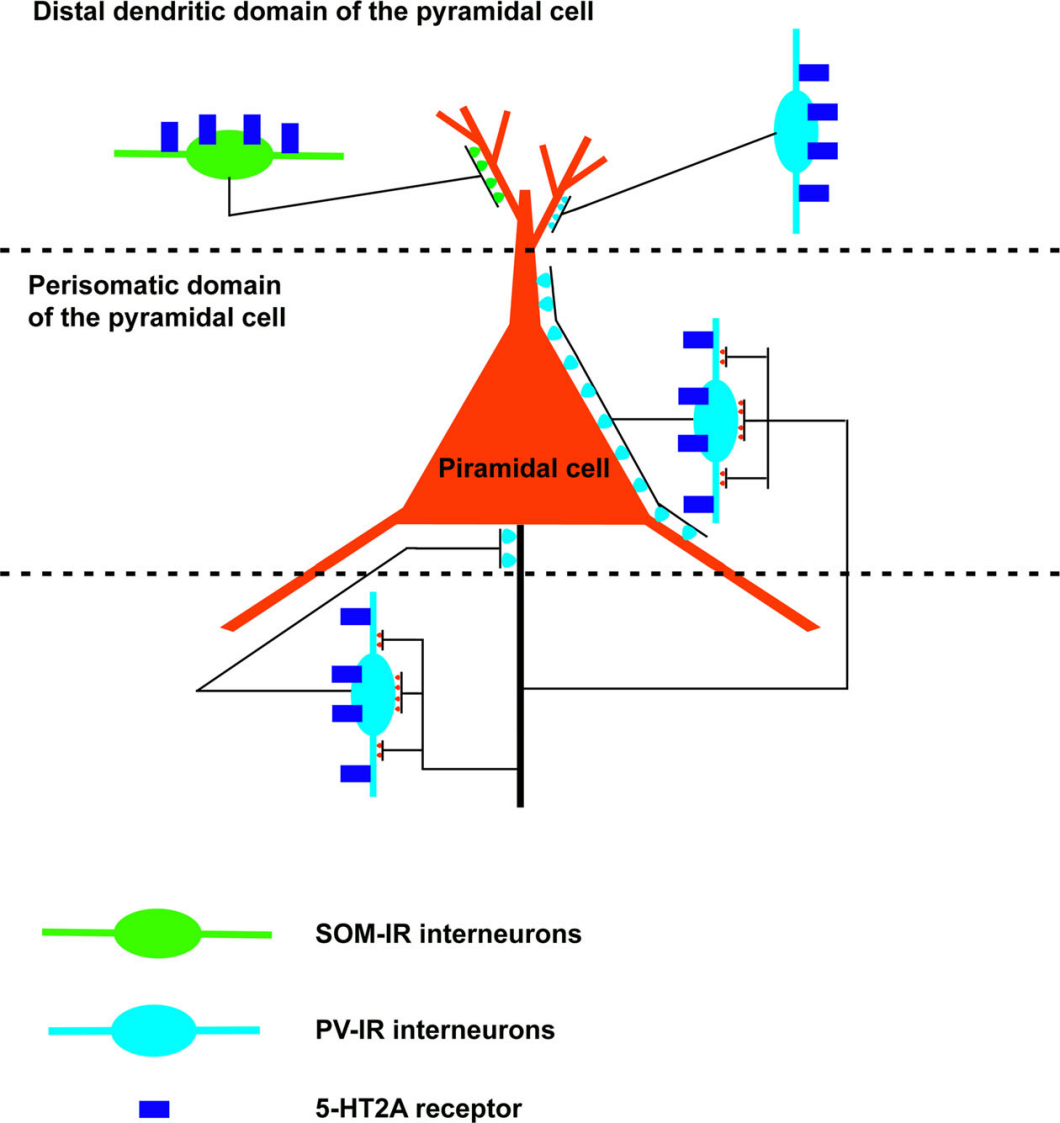
**Schematic drawing of a neuronal microcircuit expressing the 5-HT_2A_ receptor in the rat basolateral amygdala**. The 5-HT_2A_ receptor is located in excitatory (pyramidal cells) as well as inhibitory neurons. In particular, this receptor is expressed by GABAergic interneurons which innervate the initial axonal segment (parvalbumin-immunoreactive [IR] chandelier cells), the cell body and proximal dendrites (parvalbumin-IR basket cells), and the distal dendrites (somatostatin-IR cells; parvalbumin-IR interneurons) of the pyramidal cells. Note the reciprocal perisomatic connection between pyramidal cells and parvalbumin-IR interneurons (chandelier and basket cells).

The distribution of the 5-HT_2_ receptor previously reported indicates that this receptor could modulate amygdala activity acting on projection neurons (pyramidal neurons of cortex-like structures and GABAergic projection neurons of the basolateral amygdala and striatum-like structures) as well as on inhibitory interneurons (in particular, GABAergic interneurons of the cortex-like structures). The distribution of the 5-HT_2_ receptor family has been studied more extensively for 5-HT_2A_ receptor subtype, especially in the rat basolateral amygdala (Figure 3). In the microcircuits of the rat deep nuclei, the 5-HT_2A_ receptor is located on both pyramidal and non-pyramidal neurons. The non-pyramidal neurons containing this receptor express PV and SOM (McDonald and Mascagni, 2007). As previously reported, the PV–IR interneurons innervate the perisomatic domain (cell body and proximal dendrites) of pyramidal cells (Muller et al., 2006). Moreover, these interneurons are connected by gap junctions and constitute an inhibitory network which synchronizes the firing of pyramidal cells (Woodruff and Sah, 2007). Interestingly, most of the pyramidal neurons form intimate synapse-like contacts with the somata and dendrites (especially proximal dendrites) of the PV–IR interneurons (McDonald et al., 2005), in this way, constituting a reciprocal perisomatic connection which may be important in modulating the synchronized rhythmic activity associated with the formation of emotional memories (Paré and Collins, 2000; Paré et al., 2002; Rainnie et al., 2006). In the rat basolateral amygdala, SOM–IR interneurons innervate the distal dendritic domain of pyramidal cells and could modulate synaptic mechanisms related to emotional learning, including fear conditioning (Paré et al., 2002; Muller et al., 2007a). Since the 5-HT_2A_ receptor is located on PV-IR and SOM-IR interneurons, this receptor subtype could play an important role in the formation of emotional memories.

## 5-HT_2_ receptor family and amygdala-mediated behavior

The involvement of the 5-HT_2_ receptor family in numerous amygdala-mediated behavioral and physiological effects has been described in several reports. This receptor family plays a crucial role, especially in fear and anxiety. Local infusion of ketanserin (a 5-HT_2_ receptor family antagonist) induces an anxiolytic effect in the conflict test (Hodges et al., 1987). Microinjections of nefazodone (a 5-HT_2_ receptors antagonist) into the basolateral nucleus of the rat amygdala enhances the aversive responses induced by NMDA activation of the neural substrates of aversion in the inferior colliculus (Maisonnette et al., 2000). In different mouse models of anxiety, the 5-HT_2A_ receptor mediates different anxiolytic-like effects (Dhonnchadha et al., 2003a,b). Moreover, bilateral injections of ketanserin (a 5-HT_2A_ and 5-HT_2C_ receptors antagonist) into the rat basolateral/medial amygdala produces an anxiogenic profile in an elevated plus-maze (Zangrossi and Graeff, 1994).

The 5-HT_2A_ receptor is also implicated in kindling development from the rat amygdala since the subcutaneous injection of DOI, an agonist of 5-HT_2A/2C_ receptors, facilitates kindling development and reduces the number of amygdaloid stimulations necessary to obtain generalized seizures (Wada et al., 1997).

It is known that direct or indirect projections from the central nucleus of the amygdala to the paraventricular nucleus of the hypothalamus mediate a stress response. *In vivo* microdialysis studies have demonstrated that there is an increase in serotonin release in the amygdala during stress (Kawahara et al., 1993). Accordingly, the 5-HT_2A_ receptor located in the central nucleus of the rat amygdala is able to activate the hypothalamo-pituitary-adrenocortical axis (Feldman et al., 1998). Finally, in the rat basolateral amygdala, the 5-HT_2A_ receptor-mediated serotoninergic facilitation of GABAergic synaptic transmission is impaired by stress (Jiang et al., 2008).

The 5-HT_2B_ receptor is also involved in amygdala-mediated behavior. In fact, the activation of this receptor subtype causes anxiolysis in social interaction tests in the rat (Duxon et al., 1997b).

## Conclusions

The present review reported that the 5-HT_2_ receptor family plays a crucial role in regulating the activity of amygdalar microcircuits and projections. In fact, as in the cerebral cortex and the hippocampal regions (Willins et al., 1997; Hamada et al., 1998; Jakab and Goldman-Rakic, 1998, 2000; Cornea-Hébert et al., 1999; Clemett et al., 2000; Xu and Pandey, 2000; Jansson et al., 2001; Miner et al., 2003; Lüttgen et al., 2004; Bombardi, 2012), excitatory as well as inhibitory neurons in the rat amygdala express the 5-HT_2_ receptor family. Nevertheless, the exact role of the 5-HT_2_ receptor family in the modulation of amygdala activity is still poorly understood and requires additional study. In this way, detailed knowledge of the cellular mechanism underlying the modulation of amygdalar activity mediated by the 5-HT_2_ receptor family could provide valuable information for better understanding the pathogenesis of affective disorders and for utilizing a more specific pharmacological treatment.

### Conflict of interest statement

The author declares that the research was conducted in the absence of any commercial or financial relationships that could be construed as a potential conflict of interest.
